# Lessons Learned from Two Decades of Modeling the Heat-Shock Response

**DOI:** 10.3390/biom12111645

**Published:** 2022-11-07

**Authors:** Ayush Ranawade, Rati Sharma, Erel Levine

**Affiliations:** 1 Department of Bioengineering, Northeastern University, Boston, MA 02115, USA; 2 Department of Physics, Harvard University, Cambridge, MA 02138, USA; 3 Department of Chemistry, IISER Bhopal, Bhopal 462066, Madhya Pradesh, India

**Keywords:** heat shock response, mathematical modelling, heat shock proteins, heat shock factor, differential equations, sensitivity analysis

## Abstract

The Heat Shock Response (HSR) is a highly conserved genetic system charged with protecting the proteome in a wide range of organisms and species. Experiments since the early 1980s have elucidated key elements in these pathways and revealed a canonical mode of regulation, which relies on a titration feedback. This system has been subject to substantial modeling work, addressing questions about resilience, design and control. The compact core regulatory circuit, as well as its apparent conservation, make this system an ideal ‘hydrogen atom’ model for the regulation of stress response. Here we take a broad view of the models of the HSR, focusing on the different questions asked and the approaches taken. After 20 years of modeling work, we ask what lessons had been learned that would have been hard to discover without mathematical models. We find that while existing models lay strong foundations, many important questions that can benefit from quantitative modeling are still awaiting investigation.

## 1. Introduction

In recent decades, the advent of novel methods for quantifying molecules and processes in living cells drove a rapid expansion in quantitative modeling of biological systems. It is widely believed that mathematical modeling can bring new understanding to the complexity of regulatory processes and information processing, to identify governing principles, and to guide intervention and design of synthetic genetic circuits [1,2,3]. To corroborate this view, it would be informative to consider well-studied examples and ask what facilitates significant contributions from mathematical modeling, and what might impede it. Here we address this question in the context of mathematical modeling of the cellular heat-shock response (HSR).

The HSR is a universal genetic program ordained with maintaining the homeostasis of the proteome in response to unfavorable conditions [4]. Under conditions that induce protein misfolding, unfolding or aggregation, cells respond with a significant increase in the production of heat-shock proteins (HSPs). Most of these proteins act as chaperone that facilitate stabilization of proteins in their native folded state, guide their aggregation and translocation, or target them to proteolysis [4]. A functional HSR is critical for the fitness of the organism, and its dis-regulation is implicated in metabolic disorders, degenerative and inflammatory diseases, and cancer [5,6,7,8]. Despite divergence in the molecular identity of the proteins involved in the HSR and in many of the underlying mechanistic details, the design of the heat-shock regulatory program and its logic are conserved across biological kingdoms [4].

Due to its critical importance in health and disease, the HSR system has attracted much attention from researchers interested in quantitative modeling of genetic systems. The clear function of the system and the well-defined signals that trigger the HSR make it easier to ask questions and formulate hypotheses that are biologically relevant. Moreover, the similarity and differences among implementations of the HSR system in different organisms enable search for unifying principles. The HSR has been studied through quantitative modeling in multiple organisms, including bacteria, yeast, algae, worms and mammalian cells [9,10,11,12,13,14]. These models vary in their level of detail, the type of questions addressed, the conceptual framework employed, and the way experimental data have been utilized. These works were published in different formats with varying levels of accessibility to readers who lack experience in mathematical modeling.

In this paper we describe these modeling studies, the questions they address, the approaches they take, and the discoveries they make. We point out the advantages provided by formal, mathematical modeling, and consider how these are integrated into the biology of HSR. After 20 years of modeling the HSR, we ask what has been learned, and point out possible directions for the future.

## 2. The Titration Feedback Is Central to the HSR Regulation

**Figure 1:**
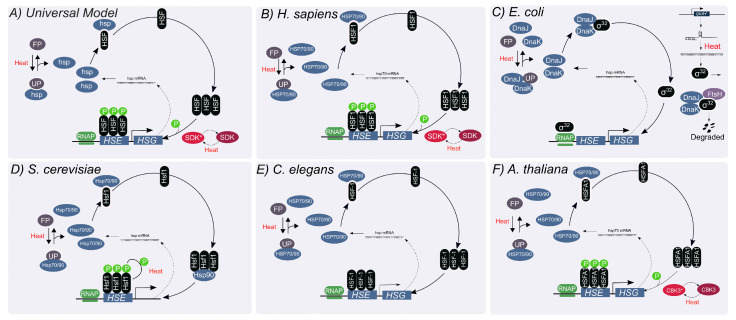
**Overview of the HSR Model.** The central element of HSR is the stress-induced activation of the heat-shock transcription factor, HSF, resulting in the initiation of heat shock proteins (HSP) synthesis. The respective core model of HSR in different model organisms is represented. UP: Unfolded protein, FP: Folded proteins, ℗: phosphorylation, SDK and CBK3: inactive protein kinase, SDK* and CBK3*: active protein kinase.

In broad stokes, the system that controls the HSR in all studied organisms can be described by a single, ‘universal’ model (Figure 1A). This model is comprised of three types of molecular components: at least one heat-shock transcription factor (HSF), several families of heat-shock proteins (HSPs) consisting mostly of molecular chaperones, and a diverse group of unfolded proteins [15]. The regulation of the distribution of HSF among different possible states—most of which are transcriptionally inactive—is the main molecular mechanism for controlling the level of HSR induction. Under un-stressed conditions, HSF interacts with HSPs to form an inhibitory complex that renders HSF inactive. Upon heat shock, an influx of unfolded proteins titrates HSPs away from HSF. The released HSFs form homo-trimers and bind to heat-shock elements (HSE) at promoter sites. The HSE-bound HSF can then be hyper-phosphorylated to enhance the expression of HSPs. Newly synthesized HSPs act as chaperones to restore proteostasis by removing unfolded proteins. Excess HSPs can interact with HSF to reform inhibitory complexes and suppress the expression of HSPs. This mechanism is commonly referred to as the chaperone-titration feedback loop [15].

### 2.1. The HSR in Mammalian Cells

Heat shock proteins have many roles in human development and health. The high molecular-weight ATP-dependent HSPs (70 kDa and above, including the abundant Hsp70) are involved in protein folding and their proper assembly and translocation, and are implicated in the surveillance against cancer and induction of immunoregulation [6,8]. The low molecular-weight proteins (10–60 kDa) act as molecular chaperones to facilitate folding and protein translocation, and are implicated in cellular proliferation during normal development and tumor growth [16].

The mammalian HSF family consists of four members, involved in multiple physiological processes including development, immunity, aging and stress response. HSF1 is recognized as the principal heat-responsive factor, with additional contribution from HSF2 [17]. As in the ‘universal’ model, titration of HSPs by misfolded proteins away from HSF1 is central to the HSR activation (Figure 1B) [18,19]. Under normal conditions, HSF1 is constitutively expressed, and found in an inactive monomeric state bound to HSP70 [20]. Upon upshift in temperature, HSF1 homotrimerize to attain an active, DNA-binding competent state, and binds HSEs to induce the transcription of HSPs [21]. The HSE-bound HSF1 is hyperphosphorylated to fully activate the synthesis of HSPs [22]. Increased concentration of HSPs sequester HSF1, resulting in a decline in their expression [20].

### 2.2. The HSR in Escherichia coli

HSR in bacteria has been studied in many species, but modeling has focused mainly on the HSR of *E. coli*. HSR of these enterobacteria is regulated by overlapping mechanisms, one of which follows the chaperone-titration feedback loop of the ‘universal’ model. Here, transcription of heat-shock proteins relies on the alternative sigma factor $\sigma^{32}$ (RpoH) because *E. coli* lacks a homolog for HSF. (Figure 1C) [23]. Binding of protein chaperones DnaK (HSP70 homolog) and DnaJ (HSP40) to $\sigma^{32}$ forms an inhibitory complex which cannot bind to the RNA polymerase [23,24,25,26]. In addition to the titration feedback loop, the total concentration of $\sigma^{32}$ is regulated at the translational and post-translational levels. The mRNA of rpoH displays a secondary structure that occludes its ribosomal binding site, thus limiting its translation. This structure melts at high temperatures, exposing the ribosomal binding site and improving the efficiency of translation of $\sigma^{32}$ [27]. This mechanism uses temperature information independently of the folding state of cellular proteins to affect the production of HSPs. In addition, $\sigma^{32}$ is rapidly degraded by FtsH, an ATP-dependent metallo-protease, through interaction with DnaK/J. This FtsH-mediated degradation feedback loop facilitates rapid removal of $\sigma^{32}$ when DnaK/J is in excess [28].

### 2.3. The HSR in S. cerevisiae

The ‘universal’ model also describes the HSR of *S. cervisiae* (Figure 1D) [29]. Under basal conditions, Hsf1 is bound in a repressor complex by chaperones [30]. Upon heat shock, chaperones Hsp70 and Hsp90, their co-chaperones and the chaperonin TRiC/CCT are titrated away by unfolded or misfolded proteins, leaving Hsf1 free to activate the transcription of chaperone genes. Once proteostasis is restored, the freed chaperones deactivate Hsf1 by forming a repressor complex. However, some aspects of the HSR regulation are yeast-specific, such as a constitutive trimerization of Hsf1, which is heat-shock responsive in mammalian cells [31]. In *S. cerevisiae* phosphorylation of Hsf1 enables differential tuning of the proteostasis network in individual cells, allowing populations to access a range of phenotypic states [10].

### 2.4. The HSR in C. elegans

Since the core mechanism of HSR in *C. elegans* follows the universal model [32], research in the worm has focused on organism-level attributes of the HSR. Elevated temperatures induce an avoidance behavior, in which worms migrate away from areas of noxious temperatures [33]. Some of the thermosensory neurons involved in this behavior have been shown to be involved in regulating activation of the cellular HSR in distal tissues [14,34,35,36]. Possible roles of systemic regulation of the HSR include distinguishing acute heat stress from chronic protein misfolding [36] and modulation of the HSR by olfactory experience [35]. In addition, a study of the expression patterns of different HSP families suggested tissue-specific programs of HSR regulation [32].

### 2.5. The HSR in A. thaliana

Plants are exposed to wide temperature changes on multiple time scale, and have no way to avoid noxious temperatures. It is therefore not surprising that the HSR in plants is highly complex and involves multiple regulatory mechanisms [37]. This complexity is exemplified by the presence of a large number of HSFs (e.g., 21 HSFs in *Arabidopsis* [38] and 26 in tomato [39]), that are divided into three classes. HSFA1s are the master transcriptional regulators or the HSF in plants, and their activation under stress occurs at multiple levels, both directly and indirectly [40]. One of these mechanisms is the ‘universal’ interaction with HSPs, which regulate its activity (HSP70) and nuclear localization (HSP90) [41,42,43]. Other HSFs act with HSFA1 or alone to modulate the response and to contribute to long-term thermotolerance [37,44,45].

Another layer of HSR regulation in plants involves a post-transcriptional control of HSFs by alternative splicing. Under prolonged heat stress, regulated splicing yields a *HSFA2* mini-exon targeted for nonsense mediated decay, used to adjust the levels of *HSFA2* mRNA according to the cellular needs [46]. Additionally, under severe stress, the mini-exon is retained in the mRNA, and its product binds to *HSFA2* promoter and cause over expression of the full-length *HSFA2*. Similar stress-dependent alternative splicing is also observed in other HSF members [40]. Furthermore, translation of HSFs is regulated by the presence of a micro open reading frame (uORF) in their 5’ untranslated regions (UTRs) [47], leading to increased suppression upon exposure to heat. The complexity of the HSF-mediated regulation is further enhanced by interactions with non-chaperone proteins (e.g., heat shock binding protein (HSBP)). For example, in *Arabidopsis*, HSBP is expressed in response to heat stress and its interaction with the HSF oligomers cause inactivation of the HSF activity [48].

## 3. Experimental Evidence of the Mechanism and Basis of Models

Experimental evidence is the foundation on which any theoretical model is built. How data are used can be categorized into two general steps. In the first step, exeprimental observations are used to recognize the molecular species involved in the system and the interactions among them. These are respectively the nodes and the connections (or edges) of the biological network under study. Biochemical and molecular studies can provide estimations of reaction rates, affinities, and concentrations, which facilitate the translation of network structures into mathematical models.

In the second step, quantitative measurements are used to calibrate, validate and test proposed models. These data can, for example, characterize input-output relations between signals and response, or provide time-series measurements of concentrations, spatial distributions, and fluxes of some of the molecular species during the operation of the system or in response to some perturbations.

In this section we describe the data used in the first of these two steps, namely, data used to establish the structure of the HSR network. These data defined the key nodes of this network—HSFs, HSPs, and misfolded proteins—and uncovered the interactions among them, predominantly the titration feedback loop. The second step of integrating data with models will be described in the next section.

**Heat-shock activation of HSF through trimerization.** Western blot analysis of HeLa cell extracts upon heat treatment showed that HSF trimerizes during a 43 °C heat shock for one hour [49], and translocates from the cytosol to the nucleus within 15 min. Inhibiting HSF production in anti-inflammatory drug-treated HeLa cell extracts by exposing them to 42 °C heat revealed that trimerized HSF is a stable intermediate which undergoes phosphorylation and initiates subsequent *HSP70* transcription [22,50]. In yeast, in contrast, phosphorylation of HSF1 is not required for its transcriptional activity, although it may enhance its activity, as revealed by experiments with mutant strains lacking its phosphorylation sites [10]. Analogous trimerization mechanism is also observed in bacteria. Transient repression of HSPs in *E. coli* was observed following a downshift from 42 °C to 30 °C even though the concentration of $\sigma^{32}$ remained elevated. In addition, overexpression of $\sigma^{32}$ leads only to a transient increase in HSPs [51]. 

**The chaperone-titration feedback loop.** Molecular events involved in the regulation of inducible HSPs revealed the titration feedback loop in human cells. Transgenic overexpression of *HSP70* in HeLa cells led to a four to five-fold repression of the transcriptional activity of the HSF activation-domain. This showed that HSPs convert HSF to a transcriptionally inert state by binding to the active transcription complex [20]. In unstressed HeLa cells, immunoprecipitation of HSP90 complexes with anti-HSF antibodies showed that HSF exists in a complex with HSP90. Just a 5 min 45 °C heat shock to these cells released HSF from the complex. Together, these data suggest that HSP prevent free HSF from activating transcription [52]. Similar mechanism has been demonstrated in yeast, where immunoprecipitation experiments revealed that Hsp70 dissociates from Hsf1 transiently (for the first 5 min) during a 39 °C heat shock [10]. Similar chaperone mediated repression is observed in bacteria. By expressing the *dnaKJ* operon from an IPTG-inducible lac promoter, it was shown that increased chaperone expression leads to lower levels and reduced activity of $\sigma^{32}$ [53]. Other molecular chaperones such as GroEL/S showed similar titration outcomes [26].

**Degradation of $\sigma^{32}$.** Bacteria is the only system in which degradation of $\sigma^{32}$ also plays a role in HSR regulation. Accumalation of the chaperone DnaK, DnaJ and GrpE induces degradation of $\sigma^{32}$ via a FtsH-dependant mechanism [25,26,53,54].

**Possible post-transcriptional regulation of HSP70**. *HSP70* mRNA levels in actinomycin treated (transcriptionally arrested) 293 (human embryonic kidney) cells were determined by S1 nuclease analysis. A 30 min 43 °C heat shock applied to these cells increased stability of *HSP70* mRNA compared to cells maintained at 37 °C. Half-life of *HSP70* mRNA in unstressed cells was estimated at 54 min, but was longer than 2 h after a 43 °C heat shock [55].

## 4. Mathematical Modeling of the Heat-Shock Response

In this section we provide a brief overview of the process of mathematical modeling a biological system, some of the choices and approaches one can take. A reader familiar with mathematical modeling may choose to skip this section.

### 4.1. From a Biological Question to a Mathematical Model

Like any valuable study, mathematical modeling of a biological system starts with a well-defined question or hypothesis. It is then developed to capture the relevant aspects of the biological system that pertain to the question of interest. Often, the question one is interested in studying dictates the mathematical framework to be used (see Section 4.2). The modeler is then faced with several important choices. First, what level of detail is to be included explicitly in the model. Such detail includes the number of molecular species and the interactions among them. For example, binding-unbinding events that occur significantly faster than other processes are often not considered explicitly, but are replaced with an “equilibrium” ratio between bound and unbound states. The modeler should also decided how to deal with spatial, temporal, and molecular inhomogeneities.

Finally, once the structure of the model has been laid down, all parameters of the model—including reaction rates, diffusion and transport coefficients, concentrations of auxiliary molecules, etc.—should be assigned. In rare cases, parameters have been measured directly in vivo in the relevant system. More often though, parameters have been measured in vitro, or in similar systems (say, an organism of the same clade or genus). In such cases, one may assume that the parameter values in the system they consider share the same order of magnitude as the ones measured elsewhere, or assume that parameters keep their ratios in the different systems, even if their absolute values are quite different.

Even for a well-studied system, the values of some parameters may be unknown. These are sometimes called ‘free parameters’. Their values may be inferred from the model, by comparing the ‘behavior’ of the model (described below) with the same behavior measured experimentally. This, however, must be done very cautiously to avoid potential pitfalls. For example, if the number of free parameters is too large (compared with the amount and complexity of the available data), the inference procedure may yield values for these parameters that do not reflect their true values, and in some cases might do so even if the model is completely wrong. In some cases—but not always—this can be avoided by checking that the inferred values are realistic and sit well with what could be expected.

**Figure 2:**
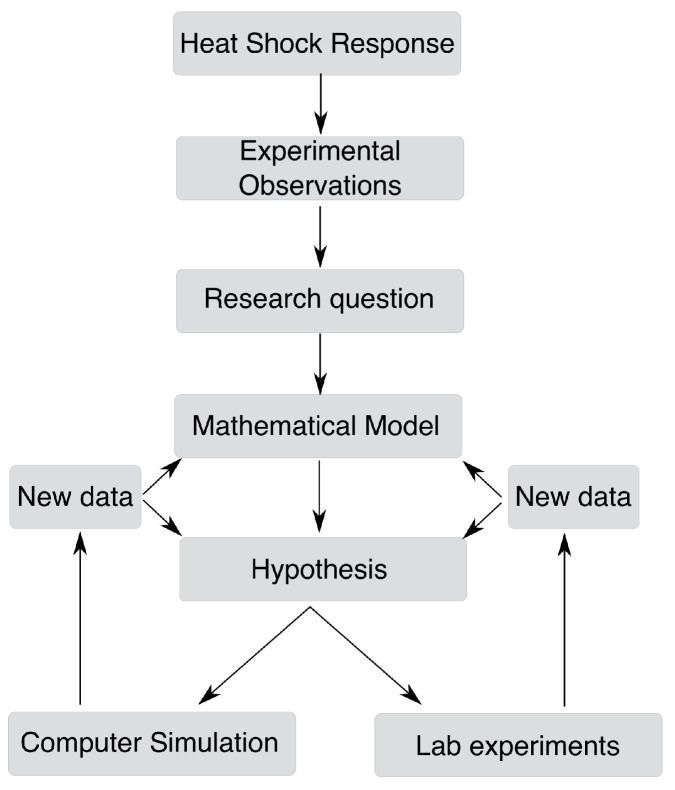
Overview of the mathematical modeling process.

With a fully defined model, one can compute different behaviors of the system to answer relevant research questions. These behaviors can refer to the steady-states of the system, the dynamics of the system following an external perturbation or noise or the mapping between any of these system properties to external signals. These predictions can then be tested experimentally to validate or refine the model. This workflow is depicted in Figure 2.

### 4.2. Alternative Modeling Frameworks

The most common framework for modeling biological systems is that of dynamical systems. In this framework, temporal changes in the concentration of different molecules are modeled as a set of Ordinary Differential Equations (ODEs), sometimes supplemented with algebraic equations (then called Differential Algebraic Equations, DAEs). These are similar in structure to chemical rate equations. Most of the models described in this review fall into this category.

**Figure 3:**
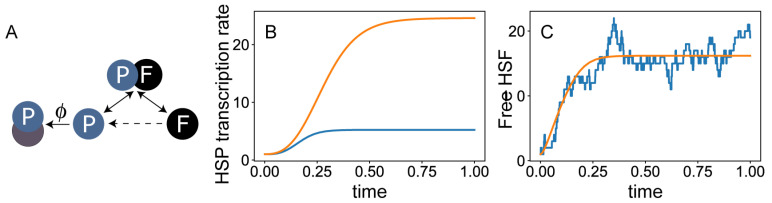
**The core of the mathematical models of the HSR.** (**A**) An overly simplified model. This model explicitly considers only two species, HSF and HSP. While HSPs are created and destroyed, it is assumed that the total concentration of HSF is conserved. (**B**) Simulation of the model with two levels of heat-shock, accounted for through the flux ϕ of titration of HSP by misfolded proteins. (**C**) Comparison between the increase in HSF concentration after heat shock as predicted by an ODE-based model (orange), and by a stochastic simulation of the model (blue).

To demonstrate this approach, consider a system of DAEs that models the titration feedback loop at the heart of the HSR (Figure 3A). In this model, we consider explicitly only a single species of HSP and the free form of HSF. The concentrations of the two are denoted, respectively, by *p* and *f*. We suppose that the total concentration of HSF is fixed, and denote this concentration $f_{\mathrm{tot}}$, which is one of the parameters of the model. Thus, the concentration of HSF-HSP complexes is given by $f_{\mathrm{tot}}-f$. The reactions we consider are binding of HSP to free HSF (which occurs with rate $\kappa^{†}$), unbinding of the two (with rate κ), degradation of a HSP (with rate λ), and its association with misfolded protein (with rate ϕ, which is temperature or stress dependent). Finally, the synthesis of heat-shock proteins depend on the concentration of HSF, and we write this rate as α(f), a monotonically increasing function of *f* that needs to be specified. This model is defined by the following two equations, and is therefore said to be two-dimensional (not to be confused with the dimensionality of the cell itself):

$$\begin{array}{cc} \frac{dp}{dt} & =\overset{\mathrm{HSP}\;\mathrm{production}}{\overset{⊓}{\alpha (f)}}+\overset{\mathrm{release}\;\mathrm{from}\;\mathrm{complexes}}{\overset{⊓}{k(f_{\mathrm{tot}}-f)}}-\overset{\mathrm{incorporation}\;\mathrm{into}\;\mathrm{complexes}}{\overset{⊓}{k^{†}fp}}-\overset{\mathrm{degradation}}{\overset{⊓}{\lambda p}}-\overset{\mathrm{titration}\;\mathrm{by}\;\mathrm{misfolded}\;\mathrm{proteins}}{\overset{⊓}{\phi p}}\\ \frac{df}{dt} & =\overset{\mathrm{release}\;\mathrm{from}\;\mathrm{HSF}:\mathrm{HSP}\;\mathrm{complexes}}{\overset{⊓}{k(f_{\mathrm{tot}}-f)}}-\overset{\mathrm{incorporation}\;\mathrm{into}\;\mathrm{complexes}}{\overset{⊓}{k^{†}fp}}\;. \end{array}$$

This model can be used to study the baseline steady state (setting ϕ to zero) and the dynamics of HSR (setting ϕ to a finite number and studying the departure from that baseline) (Figure 3B).

Unfortunately, using parameter values drawn from similar systems this model fails at recovering many experimental observation. For example, with parameter choices that lead to the known ratio between HSF and HSP during homeostasis, the model responds to heat shock slower than suggested by measurements. Indeed, this model is oversimplified, and one should consider different ways to improve it by including more details of the biological system.

Several aspects of the biological reality are not captured by ODEs, and require a more general framework. For example, since most biological processes are not instantaneous, one can explicitly account for the time delay between two sequential processes. These models are written using Delay Differential Equations (DDEs). Biological systems are naturally non-deterministic, and this can be accounted for by considering the different sources of fluctuations in the concentrations of each molecular species. This can be done either by considering a stochastic version of the differential equations (SDEs) or—more commonly—using a stochastic computer simulation (Figure 3C). Finally, biological systems are never spatially homogeneous, and may exhibit important spatial patterns both within the cell and across cells and tissues. These aspects can be addressed in the framework of partial differential equations (PDEs), the most famous of which are reaction-diffusion systems.

With a model in place, one can use different theoretical tools to address different questions. For example, the framework of control theory allows measuring the stability, controllability, and observability of the system, and assigning roles for different feedback loops in the system. Sensitivity analysis can be done to identify parameters of the system which have the strongest influence on the functional properties of the system. The theory of stochastic process can be utilized to define constraints on the accuracy and reliability of the system. Information theory can be used to estimate the accuracy at which the system can sense external signals and response to them.

## 5. Modeling the HSR

Several models have been proposed to characterize the HSR in different organisms, aiming to address a variety of questions about their design and operation. All the models we are aware of, which are described below, fall into the class of ‘minimal models’, namely models that attempt to capture all essential features of the model using the smallest possible number of dynamical variables and reactions taken explicitly into account, as described in the previous section. To do this, all models include only one species of HSF (even in species that have multiple members), a single species of HSPs (that represents all HSP families that take part in the titration feedback), and a single collective variable that describes all misfolded and unfolded proteins. Still, models range from just a few variables and equations to over 20.

A number of mathematical frameworks are often used for modeling the HSR. Of these, the most common is the mass-action framework, in which the interactions among species are taken to occur at rates proportional to the product of their concentrations. Such models are written in the form of a set of ordinary differential equations (ODEs) or a combinations of differential and algebraic equations (DAEs). Many tools are available for analyzing these models both numerically and analytically, employing for example concepts from dynamical system theory or control theory. Some studies of the HSR focused on the effect of molecular noise and fluctuations, using algorithms for stochastic simulations of their models.

In most cases, the data that drove the development of these models and their validation was composed of a time series measurements of the concentrations of one or more molecular species before, during, and after one or two heat shocks. Older studies relied mostly on biochemical methods, while more recent studies benefited from measurements of GFP expressed from heat-shock promoters, which allows for higher temporal resolution.

### 5.1. Modeling the HSR in Mammalian Cells

Modeling the HSR in mammals benefited from ample experimental data that characterized the dynamics of HSF-HSE binding and the concentrations of the HSP-HSF complex, phosphorylated HSF1, HSP and HSP mRNA, and more. Heat shock experiments—mostly in HeLa cells—explored a range of stress temperatures between 40–45 °C, and heat shock pulses ranging between 10 min and 6 h in duration [20,55,56,57,58,59,60,61]. Some experiments also looked at the dynamics during recovery from heat shock [20,60,61].

These experiments provide constraints for building relevant models. For example, during 42 °C heat shock, experiments revealed that HSF-HSE binding reaches maximum in 15 min after the temperature upshift, and goes back to basal levels around 2 h later. The HSP mRNA levels reach its maximal level around 1 h into the heat shock, and gradually returns to control levels in about 30 min during recovery. Under a persistent heat shock, HSP mRNA levels continue to rise for 6 h.

Multiple experiments attempted at characterizing the trade-off between heat-shock temperature and duration in terms of evoking the HSR and causing cellular damage. This question has been of interest in the context of cancer treatment since the 1980s, when the concept of “cumulative equivalent minutes” (CEM) was introduced as a metric to compare the effect of different stresses applied for different durations [62]. The results of such studies was typically summarized by an Arrhenius plot, showing the percentage of surviving cells as a function of the heat-shock intensity. This plot is typically biphasic, showing a moderate decline at lower temperatures and greater decline at higher temperatures. For human cells the transition between these two phases occurs close to 43 °C [62,63,64].

The earliest modeling framework we are aware of was published by Peper et al. [9]. Its main goal was to turn the postulated titration-feedback loop model of the HSR into a formal mathematical model, and verify that this model is plausible not only qualitatively, but also quantitatively. This mass-action kinetic model included the trimerization of HSF, binding of trimerized HSF to HSE and the titration mechanism, where stress-induced accumulation of unfolded proteins titrates HSPs away from the HSF-HSP complex to induce synthesis of HSPs. The model was simulated to compare the predicted HSP70 dynamics with that measured in Reuber H35 rat hepatome cells upon two 30-min 42 °C heat shocks separated by 16 h. The fact that the simulations captured the experimentally-measured dynamics was taken as validation of the model.

Several improvements to this early model were made by various groups. Rieger et al. [65] incorporated transcriptional regulation to the early model, and captured to some extent the dynamics of *HSP70* mRNA and phosphorylated HSP, previously measured in heat-shocked HeLa cells [60]. This model did not include trimerization of HSF. Two later models [66,67] accounted explicitly for more molecular processes, including transcription, translation, and trimerization of HSF, as well as transcription of HSP mRNA. These augmented models have been shown to do increasingly better in recapturing the experimental results obtained by different groups over a range of temperatures. Together, these works contribute to the level of confidence in this family of mathematical models.

Rieger et al. [68] enhanced the model to address protein aggregation. In their model, aggregation is accelerated as aggregates become larger, adding a positive feedback loop to the model. As a result, unfolded proteins are found in aggregates at lower chaperone concentration but not at higher chaperone concentration, with a possible coexistence of the two states at intermediate concentrations. This behavior can explain both the sharp transition from aggregation as the level of stress (and therefore, chaperone production) increase, and also the broad cell-to-cell variability at intermediate temperatures. An extension of the above protein aggregation model in the context of HSR also looked into the role of the HSF-HSP complex in reducing the time lag in the response to stress [69].

A more simplified model was used by Sivery et al. [70] to perform a parameter sensitivity analysis, and to systematically explore the effect of heat-shock temperature and duration on the induction of HSR. Importantly, this study identified three operating regimes of the system, depending on the heat-shock temperature (below 42 °C, between 42–44 °C, and above 44 °C).

### 5.2. Modeling the HSR in Yeast

Unlike the heat-shock elements upstream of mammalian genes, heat-shock elements on the yeast genome are continuously bound by Hsf1, even under unstressed conditions [71]. Still, like in mammals, cytoplasmic Hsf1 transiently dissociates from Hsp70 during heat shock [10]. Zheng et al. used a minimal model to explore how Hsf1 and unfolded proteins compete for binding with Hsp70. They predicted that phosphorylation positively tunes *HSF1* expression, independent of its association with Hsp70. The prediction that lowering the affinity of Hsf1 to Hsp70 should increase the HSR was validated experimentally in their follow-up work [72].

### 5.3. Modeling the HSR in Bacteria

Models in bacteria benefited from ample quantitative data, describing the changes in the concentrations of $\sigma^{32}$, DnaJ/K, and some auxiliary proteins in the hours after a heat shock [23,26,53,73]. Key feature of the HSR network in *E. coli* is the existence of multiple feedback and feedforward loops, with overlapping functionalities. The focus of significant part of the modeling work of this system was to ask what are the roles of these different regulatory arms, and to what extent they are redundant. This question has been addressed in several works within the framework of control theory [13,74,75]. This framework allowed dividing the network into functional modules, which helps explaining the roles of the different control elements. In particular, it led to the conclusion that each one of the feedback/feedforward loops has a particular role, and that together they make the HSR in bacteria sensitive, efficient, and robust.

Gene expression in bacteria is notoriously noisy, especially for genes that are weakly expressed, such as the heat-shock sigma factor $\sigma^{32}$. Modeling works from different groups verified the validity of deterministic models of the HSR in *E. coli*, and concluded that the HSR networks is particularly effective in suppressing noise [12,13,76,77].

### 5.4. Modeling the HSR in Worms

Although unicellular models (cell cultures, bacteria and yeast) allow focusing on the cellular aspects of HSR, they are incapable of addressing questions about its regulation in the context of the whole organism. Model animals, such as the roundworm *C. elegans*, offer a platform for studying the interplay between systemic and cell-autonomous response to heat shock.

Recent experiments suggest that some sensory neurons are required for the normal activation dynamics of the cellular HSR in distal tissues [34]. To model the role of two thermosensory neurons, the dynamics of GFP expressed from the promoter of the intestinal HSP-16.2 was measured in wild-type worms and in neuron-ablated mutants [14]. These data were interpreted in the context of a minimal model for the cellular HSR. First, it was observed that a time-delay between transcription and translation must be included in the model to explain the dynamics of HSR in wild-type animals. It was then noticed that to explain the dynamics in neuron-ablated mutants, it is necessary to introduce into the model a time delay between the emergence of misfolded proteins and HSR induction. Thus, the model suggested the hypothesis that thermosensory neurons enhance the sensitivity of the HSR program to misfolded proteins, and accelerate its induction.

In addition to improving our understanding of systemic and cell-autonomous response to heat shock, mathematical modeling of HSR in individual *C. elegans* identified proteostasis capacity as a key, non-genetic regulator of heterogeneity in HSR dynamics among isogenic animals cultivated in the same environment [78]. Observations in *C. elegans* and Hela cells supported this prediction where increasing protein translation capacity leads to a proportional increase in the HSR activity. Further characterization of this mathematical model of HSR dynamics in young and older nematodes predicted an age-dependent decline in HSR activity resulting from the reduction in proteostasis parameters. This prediction was subsequently confirmed experimentally, where a more robust HSR activity was measured in embryos compared to adult *C. elegans* [78]. Moreover, the model also predicted an aging-dependent increase in inter-animal heterogeneity and a decline in intra-animal variability in proteostasis and HSR capacity. Lifespan analysis of the *C. elegans* subpopulations corroborated this prediction, where nematodes displaying a higher HSR capacity in early life were shown to have a more extended lifespan [78].

### 5.5. Modeling the HSR in Plants

The dynamics of HSR in plants is not well characterized. Recent studies provide insight into the dynamics of this network in the algae *Chlamydomonas reinhardtii*, a model photosynthetic organism, challenged with one or two consecutive heat shocks [79,80].

Plants are exposed to temperature changes on multiple time scales, some of which are part of the normal daily or annual cycle, and some are abrupt and transient. Recently, a modeling work [11] has been tasked with understanding the response of the HSR network of *C. reinhardtii* to these different temperature changes. This model showed that under a slow gradual increase in temperature, which is likely to be part of the natural experience of a plant, these algae prevent accumulation of misfolded proteins without activating the HSR. In contrast, an abrupt temperature upshift leads to induction of HSR. Interestingly, this model does not include a titration-feedback loop. Instead, activation of the HSF relies exclusively on a stress-sensitive kinase.

## 6. What Did the Mathematical Models Contribute to Our Understanding of the HSR

As we’ve seen, a significant body of work was devoted to mathematical modeling of the HSR. We now ask how these works contributed to our understanding of the HSR beyond what could be interpreted directly from experimental observations. 

**Early model of mammalian HSR and its extensions to other species.** The usefulness of a mathematical model comes forth when it goes beyond a descriptive model, and presents in quantitative terms a model that explains experimental data. To our knowledge, Peper et al. were the first to write down a titration-feedback model for a mammalian cell in mathematical terms [9], and validate that this model is consistent with quantitative observations. The model was then used to explain the temporal dynamics of HSF1 and HSP70. This model served as the basis for more detailed models for the HSR in mammalian cells, as well as models of the titration-feedback mechanism in other species, such as bacteria [12] and yeast [72]. Other works confirmed the validity of these models even when accounting for the stochastic nature of biological processes [12,81]. These models identified the low copy numbers of HSF as the main source of noise in the HSR system.

**Sensitivity analysis.** Mathematical models are often used to identify kinetic parameters whose variation has a strong effect on the behavior of the system. Sensitivity analysis of the HSR in mammalian cells [65,67], bacteria [82] and yeast [72] identified the binding affinities of molecular chaperone to phosphorylated and free HSF as critical determinants of the timing and magnitude of the response. Krakowiak et al. [72] validated their predictions experimentally by creating mutant strains of yeast with a range of binding affinities between chaperones and Hsf1. These results suggest that disruptions to this binding process—for example, by mutations in the binding surfaces of either HSF or HSP—can lead to malfunction of the HSR, and suggests that this affinity can be a target for clinical interventions or synthetic biology applications.

**Necessary complexity leads to robustness.** Studies of the HSR in *E. coli* and other bacteria identified multiple overlapping feedback loops, raising questions about what their functional roles are, if any. Taking a control theory approach, El-Samad et al. [13,75] showed that the presence of multiple loops at different steps of the process are necassary to confer the system with robustness against stochastic fluctuations. This analysis allowed assigning different loops with different roles: while the feedforward module provides gain in magnitude of response, the feedback modules work to accelerate the system in reaching the optimum response.

**Operating regimes of the HSR.** Multiple modeling works [9,11,12,14,70,83,84,85] found that the HSR has multiple operating regimes, defined by distinct levels and dynamics of activation. In particular, the system displays a limited response at stress levels below a certain threshold, and a significant response above it. This raised several questions, including what mechanisms are responsible for the existence of distinct regimes, what properties of the stress signal trigger the transition from one regime to another, and what sets the threshold levels at which these transitions occur. The sharpness of the transition between one regime and another was attributed to the non-linearilties in the activation of HSF [9,85] and to the time-scale mismatch between HSF activation and HSP production [14]. The temperature of the heat shock is a clear determinant of the operation regime [70], but the threshold temperatures depend strongly on the duration of the heat-shock pulse [14,85] as well as on the steepness of the temperature increase [11].

**The steady-state concentration of HSP determines the threshold for activation.** Several models suggested that the threshold level between operating regimes is set by the concentration of HSPs under normal conditions [14,70,84]. These HSPs are sufficient to combat the effect of stress below the threshold level. Responding to the stress of higher intensity requires more HSPs, which necessitates the activation of the HSR. Similarly, in a sequence of two heat shock pulses, qualitatively different responses to the second heat shock were explained by the concentration of HSPs that remain in the cell after the first heat shock, and may or may not suffice to handle the second shock [14,83].

**The dependence of HSR properties on the level of HSF.** Most modeling works had not focused on the level of HSF as an important determinant of the HSR, apart from predicting [65,70]—and in one case validating experimentally [10]—that overexpression of HSF beyond physiological levels should counteract the titration feedback loop. One exception is bacteria, where the level of σ32 is controlled post-translationally during heat shock. Indeed, models of the HSR in *E. coli* suggested that while the transcription level of HSR has no significant effect on HSR, the post-transnational feedback loop has a role in controlling its dynamics [75].

**Regulation of HSR by phosphorylation of HSF.** The phosphorylation of HSF is another mechanism that regulates the level at which HSR is activated. Leach et al. [86] used a combination of theory and experiment on *C. albicans* to predict transient phosphorylation via regulation of a phosphatase-inhibitor pair, leading to a perfect adaptation to heat shock. They also predicted reduced HSF phosphorylation during short sequential heat shocks and maintenance of phosphorylated state during slow temperature transitions. HSF phosphorylation was also predicted and successively shown to fine-tune the HSR in yeast [10].

## 7. Discussion

It has been 20 years since the publication of the first modeling paper of the HSR in mammalian cells [9]. In these two decades, new experimental data that describe the dynamics of the HSR in increasing details has become available, facilitating refinements and generalizations of the original model. Experiments characterizing the HSR in other organisms gave rise to models of the HSR in diverse species, ranging from bacteria to plants.

**The things that are true to *E. coli* and elephants, and the things that are not.** At the core of the HSR regulation is a titration feedback, mediated by the interaction between the master heat-shock regulator and some species of heat-shock proteins. This titration feedback appears, in one form or another, in all studied species, from bacteria to plants and mammals. It is therefore not surprising that most models of the HSR share significant similarity. The differences between these models, however, shed light on the ways different organisms adapt their HSR to their specific life style and environment.

The best example of this application is the comparison between bacteria and eukaryotes. Regulation of the HSR in bacteria relies on multiple feedback and feedforward loops, that go beyond the titration model present in their eukaryotic counterparts. Models of HSR in bacteria have been used to implicate these overlapping loops in ensuring rapid and robust response that is insensitive to fluctuations. These properties are of particular importance in rapidly dividing cells, whose fitness is directly related with their growth rate, and in cases where small copy numbers of some molecular species are involved in regulation, making them sensitive to fluctuations.

If the structure of a model is adequate for describing the HSR in two different species, albeit with different sets of parameter values, then one can compare the two sets and ask how their values reflect the required functionalities of the two systems. This has been attempted in [65], which compared the mammalian HSR to that in yeast. By changing two parameters of the mammalian model, accounting for the higher affinity of yeast HSF to DNA and for the shorter cell cycle, the model recovered multiple aspects of the HSR dynamics in yeast. This application, however, requires a serious attempt to estimate the true physiological values of all model parameters. Unfortunately, this has not been the focus of most studies described above.

Similarly, most of the models we reviewed are not particularly useful for comparing organism-specific variations in the HSR network, because they are all highly simplified, ‘minimal’ models. In particular, these models assume a single species of master regulator (HSF in eukaryotes, sigma factors in bacteria), and follow explicitly only a single species of heat-shock proteins. This is despite the fact that some species have multiple HSFs, and all organisms have multiple types of heat-shock proteins, some of which interact directly with the HSF and some do not. Obviously, questions regarding the differences in numbers, roles, and localization of these molecular species cannot be addressed with models that do not take this diversity into account.

**‘Bottom-up’ and ‘top-down’ models.** Broadly speaking, one can identify two approaches to modeling, which can be described as ‘bottom-up’ and ‘top-down’. ‘Bottom-up’ models offer a condensed, simplified version of the available knowledge about the building blocks of the biological system and the interactions among them. Casting these interactions in a mathematical form, one asks how the structure and properties of the interactions affect the functionality of the system. All models described above fall into this category.

The ‘top-down’ approach is based on identifying the functional attributes of the system, and writing down abstract models that can perform this function. ‘Top-down’ models can be used to understand the physical and biochemical constraints on the biological system, to explain the diversity in implementation of similar systems within or between species, and to answer questions about the plasticity and robustness of the system. This approach has been taken in studying the HSR in Chinese Hamster Ovary (CHO) cells [85,87]. In these works, GFP expressed from the HSP70 promoter was used to measure the dynamics of HSP transcription after heat shock. A ‘reverse-engineering’ process allowed the authors to infer from these data the structure of the underlying genetic circuit, and postulate the existence of a titration feedback. This result suggests that a titration model is not only capable of generating the measured dynamics—as demonstrated by the ‘bottom-up’ models—but is also the simplest such model [87]. This modeling approach allowed the authors to identify a single parameter that uniquely characterizes the dynamics of GFP. The fact that this parameter has a non-monotonous dependence on the heat-shock intensity revealed the existance of two operating regimes of the HSR system [85].

**The use of a model in lieu of experimental data.** Most models of the HSR explicitly follow different modified forms of a protein, such as HSF that is or is not bound to DNA, HSF in monomeric or trimeric form, HSP that is bound to HSF or free to interact with misfolded proteins, etc. Since measuring these different species can be technically challenging, models can be used instead to characterize the relative abundance of each species. For example, Peper et al. tracked free HSPs years before such direct measurement became available [9]. Predicting the dynamics of unobservable molecular states can be considered to be predictions made by the model. When direct measurement of some of these species become available, they can be used to test the model and, if necessary, guide its further development. Such predictions may be particularly important if one or more of these molecular species could be a therapeutic target.

Importantly, making reliable quantitative predictions about the abundance of specific molecular species requires careful estimation of model parameters, including those that have not been measured in vivo. Although useful, this has not been the focus of most of the models reviewed here.

**The cellular HSR in the context of the whole animal.** Almost all models of the HSR we are aware of are limited in scope to the cellular HSR, and do not address how this system interacts with other systems both within the cell and in other cells and tissues. A recent transcriptome level study aimed to model the cellular response of yeast to a heat challenge found a robust metabolic response [88]. Such orchestrated change in the cellular metabolism provides a dynamical background in which the HSR functions, and may pose significant constraints on its dynamics and regulation.

Activation of the HSR program in different tissues of *C. elegans* depends on the activity and signaling of specific neurons [34,36,89]. Recently, the effect of ablating key thermosensory neurons on the dynamics of HSR in the worm intestine has been measured quantitatively [14]. To formulate a hypothesis about the effect of these neurons on the HSR in a distal tissue, the data was interpreted by asking what is the minimal change to the cellular model, that can explain the effect of ablating these neurons. It was found that the simplest assumption that explains the data, is that thermosensory neurons reduce the delay between an increase in environmental temperature, and the commencement of cellular response [14]. This study demonstrates how a model of the cellular response can also be useful for studying interactions of the HSR with other systems.

Data in mammalian cells [60] and in worms [14] show that the activation of the HSR depends on the temperature in which cells and animals have grown. The way past experience is integrated with the HSR network has not been addressed in previous modeling works, and is an avenue for future research.

The HSR pathway is known to be triggered by a variety of conditions that compromise the integrity of the proteome [4]. How these signals, separately or in combination, affect the dynamics of the HSR is not well understood. The interpretation of data that describe such interactions, which is highly complex by nature, would benefit significantly from well-established cellular models. Similarly, these models can be useful for studying the effects of the physiological state of an animal—well-fed vs. starved, healthy vs. sick, young vs. old—on its HSR.

**Clinical implications of modeling the HSR.** Mathematical modeling of biological systems has the potential to advance clinical applications, e.g., by identifying therapeutic targets or optimal timings for intervention.

A common avenue for identifying therapeutic targets is a parameter sensitivity analysis. In this framework, a model is analyzed to identify the parameters whose alterations could have the largest impact on the outcome of the studied process. This procedure has been applied to some of the HSR models discussed above. Interestingly, multiple studies in different organisms concluded that changes to the strength of binding of the HSF to HSPs, which forms the molecular basis for the titration feedback loop, have a significant impact on many aspects of the HSR, including the threshold for its activation and the rate and strength of its induction [65,67,72,82]. Together, these studies indicate that interventions that can modulate the kinetics of HSP binding to HSF have the best potential for manipulating the HSR.

Much of the interest in the HSR in mammalian cells comes from its role in protecting tumor cells during irradiation therapy. However, no model to date has directly addressed the dynamics of HSR in this context, or focused on possible implications for cancer patients.

In addition to proteostasis, HSPs also regulate adaptive immunity and apoptosis, and misregulation in their function leads to cancer, atherosclerosis, and autoimmune diseases like arthritis, multiple sclerosis, allergies, asthma, and Type 1 diabetes [8,90,91,92,93]. These attributes make them excellent therapeutic targets for treating various infectious diseases, autoimmune disorders, and cancers [90]. For example, pathogen-specific HSPs are used as antigens in vaccines against tuberculosis, toxoplasmosis, and other microbial infections and adjuvant arthritis [91]. Furthermore, HSP-based anticancer vaccines and chemical inhibitors, geldanamycin, are tested to treat chemotherapy-resistant cancers [90,94]. Recently, HSPs were identified to protect against COVID-19 infection and are being investigated as a potential intervention target to alleviate COVID-19 complications [95].

**Outlook.** Despite its conceptual simplicity, many questions remain about the control of the HSR, many of which can—and should—be included in future modeling studies. Understanding of how the HSR works in a living organism requires better understanding of the interactions of cellular HSR with other systems (such as the immune system or the nervous systems), the physiology of the organism, and the co-occurrence of other signals in the environment. Even inside the cell, many cellular processes—including translational inhibition [96], PolII pausing at promoters of stress-related genes [97], emergence of stress granules [98], DNA compaction during stress [99], and protein aggregation due to aging [17]—are expected to interact with the HSR. Finally, established models of the HSR can be merged with models of evolution dynamics to study its evolutionary role – including emergence of thermotolerance [100,101,102,103], adaptation to global environmental changes [11,104], as well as bridging proteomic plasticity and stability [105,106,107].

## Data Availability

No new data were created or analyzed in this study. Data sharing is not applicable to this article.
